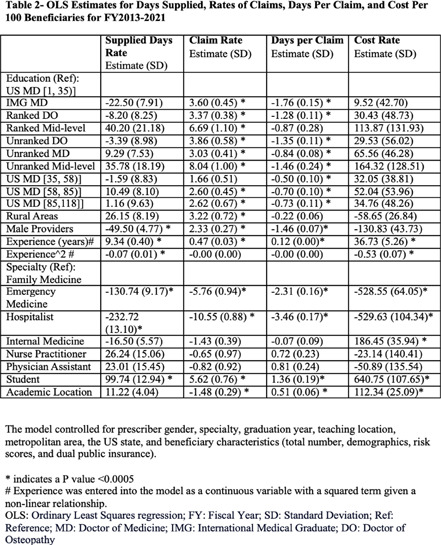# Medical School Ranking & Provider Outpatient Medicare Part D Claims for Antibiotics Among Older Patients in the US

**DOI:** 10.1017/ash.2025.194

**Published:** 2025-09-24

**Authors:** Mayar Al Mohajer, David Slusky, David Nix, Catia Nicodemo

**Affiliations:** 1 Baylor College of Medicine; 2 University of Kansas; 3 University of Arizona College of Pharmacy

## Abstract

**Background:** The overuse of antimicrobials contributes to the development of antibiotic resistance, the development of Clostridioides difficile infections, and increased patient morbidity and mortality. The impact of U.S. News medical school ranking on provider antimicrobial prescription is largely unknown. Our study aimed to assess whether there was a relationship between graduating from higher-ranked medical schools and the rate of prescribing antibiotics among Medicare Part D providers in the US. **Methods:** The ecological study obtained data from the Medicare Part D Prescribers (FY2013-2021) and the Doctor and Clinicians National repositories. The study’s main outcome was antibiotic days supplied per 100 beneficiaries. Secondary outcomes included antibiotic claims per 100 beneficiaries, days per claim, and antibiotic cost per 100 beneficiaries. A regression model was fitted to assess the relationship between provider medical school ranking and study outcomes. The study controlled for several state, provider, and patient variables. **Results:** A total of 197,540 providers were included (Table 1). No association was found between the medical school ranking and the rate of antibiotics days supplied per 100 beneficiaries (Table 2, Figure). Instead, the type of provider is associated with the prescription rates. Hospitalists and Emergency Medicine providers had fewer days supplied per 100 beneficiaries than Family Medicine providers. In contrast, students, more experienced providers (>20 years since medical school graduation), and females had more days supplied per 100 beneficiaries. Higher-ranking medical schools [1, 35], EM providers and hospitalists (vs. FM), and academic locations had lower claim rates per 100 beneficiaries, while students and experienced providers had higher claims. Days per claim were higher among providers from higher-ranked medical schools, more experienced providers, students, and academic locations, whereas they were lower among males, EM Providers, and Hospitalists. Costs per 100 beneficiaries were higher among students, academic locations, IM providers, and males; however, it was lower among EM and hospitalists. **Conclusion:** Our study showed no impact of medical school ranking on the overall rate of outpatient antibiotic prescriptions among Medicare Part D providers. While the claim rate per 100 beneficiaries was lower among providers from higher-rank medical schools compared to other providers, claims were prescribed longer, leading to similar days supplied and costs compared with other providers. This highlights the need for robust outpatient stewardship interventions and incorporating an outcome-based approach to antibiotic stewardship curricula in medical and mid-level provider schools.

**Figure:**
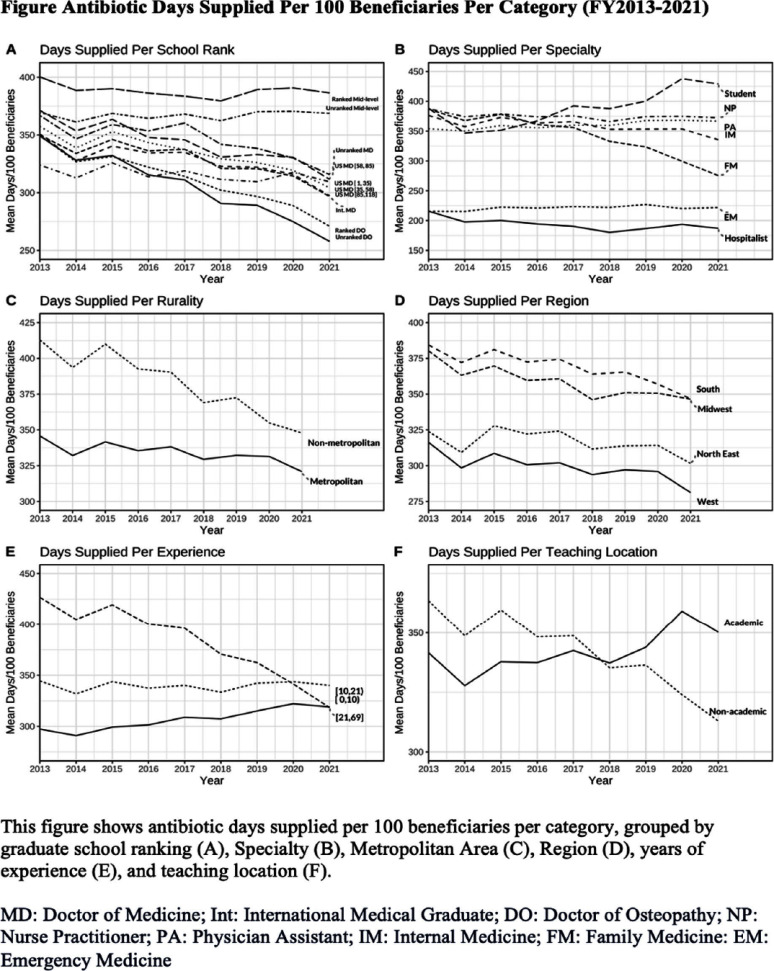

**Figure:**
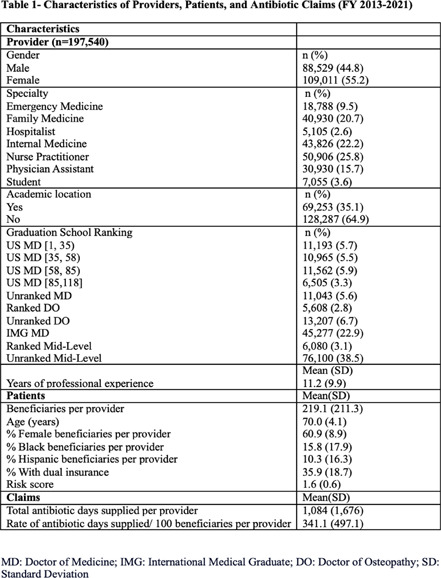

**Figure:**